# Leveraging Signatures of Plant Functional Strategies in Wood Density Profiles of African Trees to Correct Mass Estimations From Terrestrial Laser Data

**DOI:** 10.1038/s41598-020-58733-w

**Published:** 2020-02-06

**Authors:** Stéphane Takoudjou Momo, Pierre Ploton, Olivier Martin-Ducup, Romain Lehnebach, Claire Fortunel, Le Bienfaiteur Takougoum Sagang, Faustin Boyemba, Pierre Couteron, Adeline Fayolle, Moses Libalah, Joel Loumeto, Vincent Medjibe, Alfred Ngomanda, Diosdado Obiang, Raphaël Pélissier, Vivien Rossi, Olga Yongo, PREREDD Collaborators, Yannick Bocko, Noël Fonton, Narcisse Kamdem, John Katembo, Henriette Josiane Kondaoule, Hervé Martial Maïdou, Géraud Mankou, Michel Mbasi, Thomas Mengui, Gislain I. I. Mofack, Cynel Moundounga, Quentin Moundounga, Lydie Nguimbous, Norberto Nsue Ncham, Francisco Ondo Meye Asue, Yvon-Patrick Senguela, Lionel Viard, Louis Zapfack, Bonaventure Sonké, Nicolas Barbier

**Affiliations:** 1 Plant Systematic and Ecology Laboratory (LaBosystE), Department of Biology, Higher Teachers’ Training College, University of Yaoundé I, P.O. Box 047, Yaoundé, Cameroon; 2 AMAP, Univ Montpellier, IRD, CNRS, INRAE, CIRAD, Montpellier, France; 3 UGent-Woodlab, Laboratory of Wood Technology, Department of Environment, Ghent University, Coupure Links 653, B-, 9000 Gent, Belgium; 4 University of Kisangani, Democratic Republic of Congo, Kisangani, Republic of Congo; 5 Gembloux Agro-Bio Tech, University of Liège, Gembloux, Belgium; 6 University of Marien Ngouabi, Brazzaville, Republic of Congo; 7 Commission des Forêts d’Afrique Centrale (COMIFAC), Yaoundé, BP 20818 Cameroon; 8 Institut de Recherche en Ecologie Tropicale (IRET/CENAREST), BP 13354 Libreville, Gabon; 9 INDEFOR-AP, Malabo, Equatorial Guinea; 10 University of Bangui, Bangui, Central African Republic; 11 RU Forests and Societies, CIRAD, Yaoundé, Cameroon; 12 ONFi, Yaoundé, Cameroon; 13 COMIFAC, Yaoundé, Cameroon; 14 University of Yaoundé 1, Yaoundé, Cameroon

**Keywords:** Plant ecology, Climate-change ecology

## Abstract

Wood density (WD) relates to important tree functions such as stem mechanics and resistance against pathogens. This functional trait can exhibit high intraindividual variability both radially and vertically. With the rise of LiDAR-based methodologies allowing nondestructive tree volume estimations, failing to account for WD variations related to tree function and biomass investment strategies may lead to large systematic bias in AGB estimations. Here, we use a unique destructive dataset from 822 trees belonging to 51 phylogenetically dispersed tree species harvested across forest types in Central Africa to determine vertical gradients in WD from the stump to the branch tips, how these gradients relate to regeneration guilds and their implications for AGB estimations. We find that decreasing WD from the tree base to the branch tips is characteristic of shade-tolerant species, while light-demanding and pioneer species exhibit stationary or increasing vertical trends. Across all species, the WD range is narrower in tree crowns than at the tree base, reflecting more similar physiological and mechanical constraints in the canopy. Vertical gradients in WD induce significant bias (10%) in AGB estimates when using database-derived species-average WD data. However, the correlation between the vertical gradients and basal WD allows the derivation of general correction models. With the ongoing development of remote sensing products providing 3D information for entire trees and forest stands, our findings indicate promising ways to improve greenhouse gas accounting in tropical countries and advance our understanding of adaptive strategies allowing trees to grow and survive in dense rainforests.

## Introduction

Terrestrial plants account for 83% of the living carbon on Earth^1^, of which tropical forests are estimated to account for close to half^2^, principally contained within woody plant parts. Tropical forests are therefore becoming a key element in international carbon trading schemes despite obvious difficulties in accurately estimating stocks and fluxes at relevant scales, e.g., at the stand, landscape or region level. Aboveground biomass (AGB) and the associated carbon content are generally assessed indirectly via allometric models based on simple tree measurements such as trunk diameter at breast height (DBH), total height or species average mean wood density values obtained from global databases^3^. Allometric models are calibrated through destructive tree harvests requiring enormous amounts of fieldwork to cut down and weigh every tree compartment. Despite the growing number of studies aimed at calibrating allometric equations^4–6^, only a few thousand tropical trees have been sampled for this purpose so far, including very few large trees despite their specific architecture^7^ and disproportionate contribution to forest AGB^8^. This destructive method therefore offers limited promise for providing representative allometric equations for the estimated three trillion trees on Earth^9^. Terrestrial LiDAR scanning (TLS) has recently emerged as a nondestructive method not only for calibrating allometric biomass models^10,11^ but also for performing measurements of volume, and by conversion biomass, at the stand level^9^. TLS is now being promoted by the Intergovernmental Panel on Climate Change (IPCC) in its Good Practice Guidelines (GPG) for national greenhouse gas inventories, as tropical countries aim to implement more efficient and precise methodologies^12^. However, as we gain in precision and sample size with TLS, which will provide massive datasets of detailed tree volume data, there is also need for renewed care in using reliable WD values for the conversion of these volumes into AGB.

Wood performs different functions in trees such as providing mechanical support and conducting and storing water, nutrients and carbohydrates^13^. Wood-specific gravity (WSG, *sensu*^14^), or wood density (WD), has emerged as a consensus integrative trait of diverse wood properties. It is positively correlated with mechanical strength and stiffness^15–17^ and negatively correlated with water storage and capacitance^18–20^. WD mainly depends on the proportion and morphology of fibers and to a lesser extent on the proportion of parenchyma cells and morphology of vessels, which can vary between and within species and individuals^13,21–25^. With 10-fold variation across tropical tree species^13,26–28^, WD has been shown to reflect differences in regeneration guild^29–31^: pioneers tend to exhibit light wood associated with faster growth and a shorter lifespan, while shade-tolerant species invest in dense wood associated with long-term resilience. In addition, WD varies within individual trees both radially (from the pith to the bark) and vertically (from the stump to the crown top). WD radial gradients have been documented in several climatic zones, forest types and regeneration guilds^28,32–38^. While pioneer or light-demanding species exhibit steep increasing radial gradients^32^, slight increasing or decreasing radial gradients are observed in shade-tolerant species^35,37^. We may expect some similarities between radial and vertical patterns^39^ as wood is produced through the accumulation of cone-shaped volumes. Indeed, primary (vertical) growth precedes secondary lateral growth, and outer wood will exhibit the same age as wood closer to the pith higher up along the trunk or branch. However, there is currently little to no available information on the vertical variation in WD, particularly in highly diverse tropical rainforests^40–42^.

In AGB estimates, WD values are typically extracted from databases documented using samples taken at the tree base or from sawmill logs. These samples rarely if ever account for variation in WD within trees^43^. Since TLS tree volume is directly multiplied by WD to obtain an AGB estimate, any bias in WD will be directly propagated in AGB estimates (average 9% overestimation of AGB estimates at the scale of 1-ha forest stands using tree basal WD, see Sagang *et al*.^41^). As it is logistically challenging to sample WD and its vertical variation for each tree during forest inventories or LiDAR scanning campaigns, there is a critical need for a better understanding of vertical variations in WD across a wide range of tree species to improve AGB estimates.

Here, we use a unique destructive dataset collating six sampling sites in different countries and contrasted types of *terra firme* forests across the Congo basin^5^ to determine the vertical variation in WD among 822 individuals (sampled across size classes) representing a total of 51 tropical tree species; we examine possible evolutive or functional drivers and implications for AGB estimation from volumetric (TLS) data; and we propose a way to correct the aforementioned bias in a cost-efficient way.

## Characterizing WD Vertical Profiles

**Figure 1:**
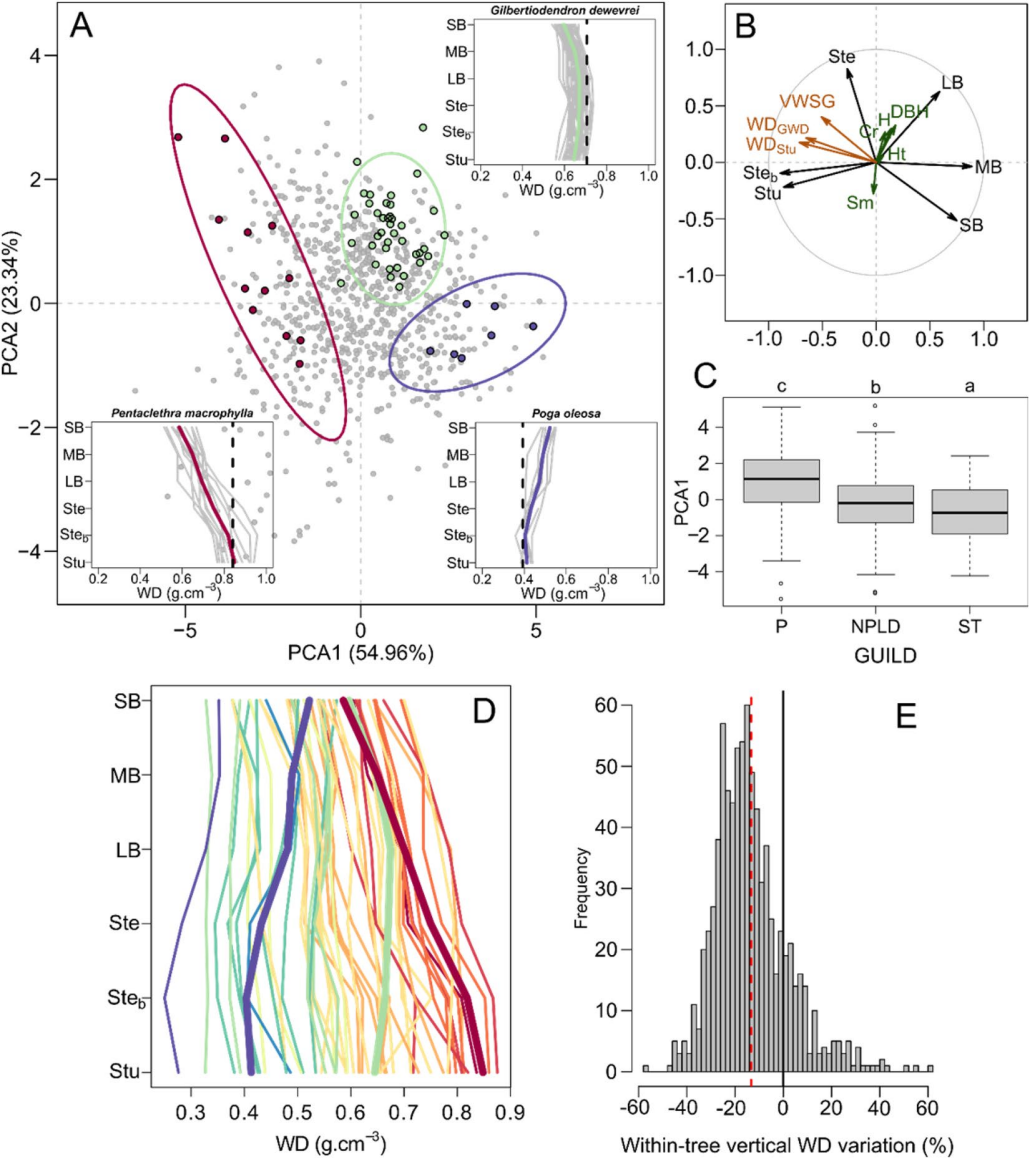
Ordination of wood density (WD) vertical profiles based on principal component analysis. Panel A: Scatter plot of PCA scores on the first two principal axes. Insets showing vertical WD profiles are highlighted for three contrasting species, with vertical dashed lines representing species mean WD derived from the Global Wood Density database. Panel B: Correlations between the WD of each tree compartment (black arrows) and the first two PCA axes. Supplementary variables related to wood density and tree structure are plotted in maroon and dark green, respectively (see Table 1 for acronyms and definitions). The histogram of eigenvalues is provided in Fig. S1. Panel C: Boxplot of PCA1 scores of individual trees by life strategy guild (pioneer: P, nonpioneer light-demanding: NPLD and shade tolerant: ST). The post hoc Tukey’s HSD test is indicated (p-value < 0.01, see Table S2 for details). Panel D: Species mean WD by tree compartment. The warm-to-cold color gradient represents the species mean PCA1 score. The three focal species from panel A are shown with thicker lines. Panel E: Histogram of tree WD differences between the small branches and the stump as the percent of the stump WD (n = 822). The dashed red vertical line represents the distribution mean.

We first compared the vertical variation in WD between 822 individual trees by determining WD in six compartments (stump: Stu, stem base: Ste_b_, stem: Ste, large-: LB, medium-: MB and small-sized branches: SB, Fig. 1D). The sample covered a broad range of WD values from 0.217 to 1.020 (Ste_b_). As there are no strong assumptions regarding the mathematical form of these variations, we used a scaled principal component analysis (PCA) to evaluate relative vertical gradients in WD. Prior to the analyses, the WD values of each tree compartment were normalized by the individual mean WD (Fig. 1A). The first PCA axis (PCA1, Fig. 1B) distinguished the tree base (stump and stem base) from the tree crown (large-, medium- and small-size branches), while stem relative WD was positively correlated with PCA2. Therefore, trees with negative scores on PCA1 exhibited a relatively high stump WD (WD_Stu_) and a decreasing WD toward the smallest branches (e.g., *Pentaclethra macrophylla*). With increasing scores on PCA1, the relative vertical profile of WD became progressively convex (for high PCA2 values, e.g., *Gilbertiodendron dewevrei*) or constant (for low PCA2 values) and eventually increased toward the small branches (e.g., *Poga oleosa*). For 83% of the sampled trees, WD was lower in the small branches than in the stump by an average of −13.3%, with individual differences ranging within approximately ±60% (Fig. 1E). When averaging gradients at the species level, a vertical decrease was observed in 88% of the species, with a range of ±30%. Species PCA scores and other characteristics are summarized in Suppl. Table 1.

### Identifying correlates of WD vertical profiles

The relative vertical profiles in WD were strongly correlated with tree basal WD, with a significant correlation coefficient (r = −0.71) between individual PCA1 scores and WD_Stu_ (Table 1 and Fig. S1A). This correlation was also observed at the species level using species-level WD obtained from a Global Wood Density database^13,44^ (WD_GWD_, r = −0.65, Table 1 and Fig. S1B). This relationship is similar to that previously reported by Hietz *et al*.^37^ in the case of radial WD gradients [Panamanian moist forest and Ecuadorian rain forest, 304 species] and Sagang *et al*.^41^ for vertical gradients [semideciduous forest of Cameroon, 15 species]. We observed a shift in the vertical WD profile from decreasing (negative PCA1 scores) to increasing (positive PCA1 scores) as species WD_GWD_ decreased (vertical dashed lines in insets in Fig. 1A). The second PCA axis was correlated with tree structure variables (Table 1, Fig. 1B). We leveraged these correlations to derive models allowing us to predict and account for vertical WD gradients (see Table 2).

**Table 1:** Tree wood and structure parameters used as supplementary variables in the principal component analysis (PCA) and their correlations with the first two PCA axes.

Parameter	Definition	PCA1	PCA2
WD_Stu_	*WD* of the stump compartment	−0.71 (***)	0.17 (***)
WD_GWD_	Species average *WD* derived from the Global Wood Density database	−0.65 (***)	0.23 (***)
VWWD	Volume-weighted tree wood specific gravity	−0.51 (***)	0.4 (***)
DBH	Diameter at breast height	0.17 (***)	0.32 (***)
H	Total height	0.08 (*)	0.27 (***)
Ht	Trunk height	0.06 (ns)	0.05 (ns)
Cr	Crown radius	0.13 (***)	0.29 (***)
Sm	Stem morphology, defined as the ratio of trunk height over tree height	−0.07 (ns)	−0.24 (***)

The probability values of Pearson correlation (r) tests are provided between brackets and coded as follows: ***P ≤ 0.001, **P ≤ 0.01, *P ≤ 0.05, ns = nonsignificant.

**Table 2:** Prediction models of whole-tree WD estimation (VWWD, in g.cm^−3^).

Models	Parameters				Performance				
	a	b	c	d	R²	RSE	AIC	B	CV
(m_1_) VWWD = a + b*WD_Stu_	0.07842 (0.00682)	0.78915 (0.01076)			0.87	0.049	−2629.1	0.86	14.8
(m_2_) VWWD = a + b*WD_Stu_ + c*DBH	0.05455 (0.00679)	0.78326 (0.01011)	0.00048 (0.00005)		0.88	0.046	−2732.3	0.75	15.2
(m_3_) VWWD = a + b*WD_Stu_ + c*DBH + d*Sm	0.10013 (0.01077)	0.77299 (0.01012)	0.00042 (0.00005)	−0.05819 (0.0108)	0.89	0.045	−2759	0.74	15
(m_4_) VWWD = a + b* WD_GWD_	0.1721 (0.00763)	0.63638 (0.01189)			0.78	0.063	−2200.6	1.41	21.8
(m_5_) VWWD = a + b*WD_GWD_ + c*DBH	0.13406 (0.00783)	0.63614 (0.01105)	0.00067 (0.00006)		0.81	0.059	−2320.9	1.19	23.8
(m_6_) VWWD = a + b*WD_GWD_ + c*DBH + d*Sm	0.18233 (0.01325)	0.62656 (0.01113)	0.0006 (0.00006)	−0.06258 (0.01395)	0.81	0.058	−2338.9	1.18	23.7

The models are based on species average WD extracted from the Global Wood Density database (*WD*_*GWD*_ in, g.cm^−3^), individual tree WD from stumps (WD_Stu_, in g.cm^−3^) and tree structure parameters (stem DBH in cm and the stem morphology index, Sm). Model coefficients are provided along with standard errors (in brackets). All coefficients are highly significant (P < 0.001). Model performance is characterized using classical fit metrics for model residuals (R², RSE, AIC), the measure of bias (B) and total error (coefficient of variation, CV) computed for AGB estimations (see the Methods section).

### Taxonomic, geographical and functional grouping

Using PCA scores to describe the relative vertical profiles in the WD of individual trees, we examined whether these profiles were clustered taxonomically, geographically or functionally. This is indeed of fundamental importance to understand the possible adaptive meaning of the profiles as well as their effect on AGB estimation bias at the stand or regional level (if similar profiles tend to be clustered at these scales).

Individual trees from the same species showed consistent relative vertical profiles in WD. For instance, 81.1% of trees exhibited the same sign along PCA1 as their species average. Similarly, 88% of the individuals presented the same sign as their species average for the difference between the tree stump and small branches. Our dataset includes only two genera with more than one sampled species, so we could not evaluate profile aggregation at the genus level. However, we found clear taxonomic overdispersion of tree PCA1 scores at the family level (K_Blomberg_ = 0.132, P = 0.014, see methods), suggesting less similarity than expected by chance at the family (i.e., strong interfamily variation) or higher taxonomic level. This finding is consistent with the phylogenetic aggregation patterns reported for WD, with decreasing similarity in WD from species to genus and low similarity in WD at the family level^26,45^.

The tree sampling site had little influence on the relative vertical profile in WD. We used ANOVA to partition the variance of tree PCA1 scores between species, sampling sites and their interaction. Since many species were found at one or two sampling sites only (28 and 14 species, respectively), we restricted the analysis to species found in at least three sites (representing a total of 330 trees from 9 species). The species effect captured 44% of the variance (F = 32.12, P < 0.001), but both the main site effect (1.8% of the variance, F = 2.20, P = 0.056) and its interaction with species (5.9% of the variance, F = 60.6, P = 0.056) were non-significant. In other words, the interspecific variation in WD vertical profiles is much greater than the site effect on a given species’ profile.

At the community level, the abundance of decreasing, stable and increasing profiles within the community is likely to change over broad regional extents given the known patterns of mean (community-level) WD variation^13^. The mean community vertical WD profile will also vary at the landscape scale following the history of forest perturbations. Grouping species into (*a priori*) regeneration guilds^46^ (Suppl. Table 1) indeed distinguished pioneer species (P) from nonpioneer light-demanding (NPLD) and shade-tolerant (ST) species, particularly along PCA1 (F = 32.5, P < 0.001, Fig. 1C). Pioneer species characterized by light wood tended to exhibit increasing relative vertical WD profiles, whereas (dense wood) shade-tolerant species exhibited decreasing relative vertical profiles. NPLD species presented intermediate scores and, hence, less contrasting profiles. This result, coupled with existing knowledge on radial WD variation in the trunk, highlights clear homology between radial and vertical WD variations and their relations to plant strategies^37^. Across the 51 studied species, the differences in species average WD were highest at the tree base and decreased toward the small branches (Fig. 1E). The broader range of species WD found at the trunk base may reflect differences in canopy accession strategies in early tree developmental stages^17^. We can think about radial and vertical WD increases in light of the costs and benefits of investing in low vs high WD. For a given biomass investment, theory predicts that building a thicker trunk with a low WD is more efficient for carrying a tree’s own weight (resistance to buckling) and resisting wind forces than building a thinner trunk with a high WD^47,48^. Although low-WD stems require less biomass investment per unit stem volume^48^, they imply higher maintenance costs of living tissues (inner bark and wood parenchyma)^49^, suggesting that WD may eventually have to increase (both upward and outward) in older pioneer trees. Narrow stems with a relatively higher WD in young shade-tolerant trees may allow greater resistance against falling branches from dominant trees^26,45^. This investment in dense wood might not be needed later in the ST tree life, which could then produce lighter outer wood. The decreasing outward WD gradient can then be reinforced during heartwood formation^35^. In mature, reiterated tree crowns, on the other hand, most trees reaching the canopy will tend to be subjected to similar biomechanical constraints when extending branches laterally. This may explain why WD then converges to a narrower range of values across all the sampled species. In other words, whereas contrasted strategies exist in early life stages, there seem to be a convergence in the mechanical or maintenance constraints met by adult trees, which is reflected by similar wood densities across species both in the branches (vertical gradients) in in the outer wood (radial gradients). This result is also congruent with the fact that large reiterated trees tend to display similar forms^50^, independent of discrepancies in the architectural model^49^ observed at the sapling stage.

### Influence of vertical variation in WD on tree AGB estimates

We leveraged volumes estimates from both (i) the 822 destructively sampled individuals and (ii) terrestrial LiDAR scanning data for a subset of 58 individual trees^10^.

**Figure 2:**
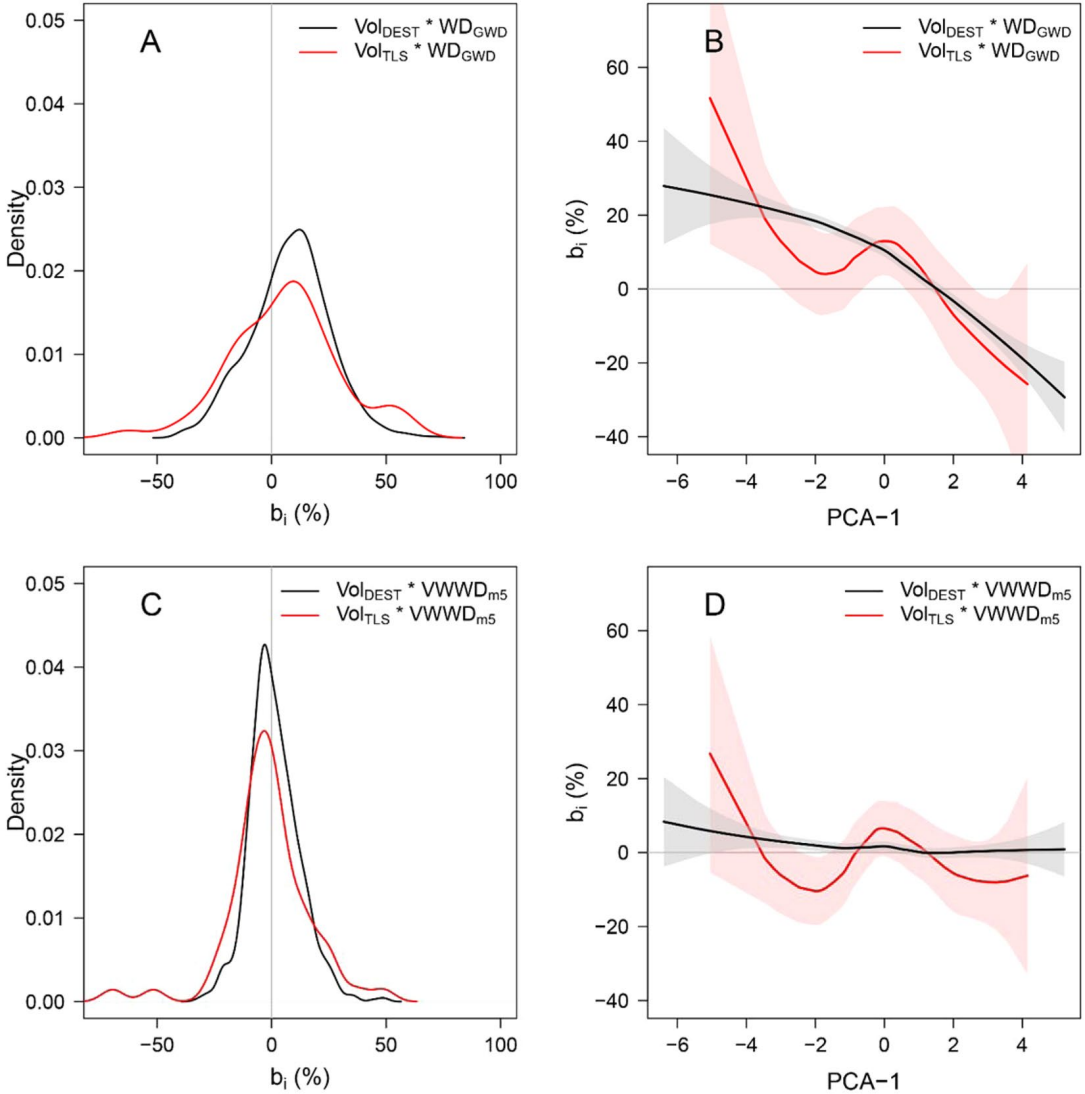
Bias in volume to mass conversion due to vertical WD gradients induced by the use of WD_GWD_: (**A**) Density plot of the relative errors (*b*_*i*_) in tree biomass estimation computed from combinations of tree volume (destructive data: black line, n = 822; LiDAR data: red line, n = 58) and species mean wood density extracted from the Global Wood Density database (*WD*_*GWD*_). (**B**) Local regression (loess function) representing the relationship between tree relative vertical WD profile (characterized by tree PCA1 score) and *b*_*i*_ of panel A. Bias is reduced when using a tree-level estimate of WD, the volume-weighted wood density (*VWWD*_*m*5_): (**C**) Density plot of *b*_*i*_ for tree biomass estimation computed from combinations of tree volume and the predicted *VWWD* from *WD*_*GWD*_ and tree DBH (model 5, see Table 2). (**D**) Local regression (loess function) representing the relationship between the relative vertical WD profile and *b*_*i*_ of panel C.

Using species WD_GWD_ to convert volumes to masses in our 822 sampled trees, we found a positive mean error across all trees (*B* = 8.12% ± 17.20%) with large individual variations (i.e., tree-level errors, *b*_*i*_, ranging from −40.31% to +72.77%; back line in Fig. 2A) and a coefficient of variation (CV) of 30.9%. Using WD_Stu_, we obtained a similar mean error across all trees (B = 8.97% ± 11.08%), with a narrower range of individual errors (from −24.05% to 61.69% and CV = 19.8%, Fig. S3A). We found similar error patterns when tree volumes were derived from terrestrial LiDAR data based on 58 scanned trees (see red line in Fig. 2A). The error in individual tree AGB estimation (*b*_*i*_) was correlated with the tree vertical WD profile (Figs. 2B and S3B). This resulted in (i) overestimation of AGB (positive *b*_*i*_) for trees with negative PCA1 scores (i.e., decrease in WD from the stump to small branches) and (ii) underestimation of AGB (negative *b*_*i*_) for trees with positive PCA1 scores (i.e., increase in WD from the stump to small branches), regardless of the WD source (i.e., WD_GWD_ or individual WD_Stu_). It is worth noting that vertical variation in WD may also affect the accuracy of AGB allometric models. For instance, we observed a similar pattern of error on AGB estimations obtained from a pantropical model^4^, with a decrease in mean tree error as PCA1 score increased (Fig. S5). This suggests that within-tree WD variation is not well accounted for even in reference pantropical allometric models.

The implications for stand-level error propagation in AGB estimation were not specifically studied here, but decreasing vertical variation in WD tended to be more frequent, explaining the overall mean positive bias obtained across our destructive (c. 9%) and terrestrial LiDAR (c. 11%) datasets and the 9% overestimation at the stand level found by Sagang *et al*.^41^ with a subset of our data. Since the vertical profile is correlated with species mean WD, and the latter value is close to 0.6 g cm^−3^ in wet tropical forests overall^44^, we may expect dominance of decreasing vertical WD patterns (see Fig. 1D) in this ecosystem. However, this may not be the case in young secondary rainforests, where the abundance of pioneer species may cancel out or even invert vertical WD patterns at the stand level. The successional trend may be different in drier vegetation, where early successional species exhibit relatively denser wood, possibly to resist higher stress levels^51^. This potential scenario has implications when estimating stand-level carbon content (emission factor) using volumetric data^12^, notably in the context of national forest or greenhouse gas inventories. We indeed expect the bias in AGB estimation to be strongly structured in space, varying with the species distribution and forest disturbance history.

### Predicting and correcting vertical WD variations

Reducing systematic errors in AGB estimations when converting tree volumes to masses requires the use of WD values that are representative of tree-level wood density, which involves integrating vertical variation in WD. For each tree, we weighted each compartment’s WD by its volume to calculate the compartment volume-weighted WD, which were summed to obtain the tree level VWWD (see Methods). We then developed linear prediction models for VWWD using easily accessible variables characterizing WD (i.e., WD_Stu_, WD_GWD_) and tree structure (tree DBH and the stem morphology index, *Sm*, used in Sagang *et al*.^41^). Prediction models based on WD alone explained most of the variance in tree VWWD (R² = 0.87 for m_1_ based on *WD*_*Stu*_ and 0.78 for m_4_ based on species average WD_GWD_, Table 2). Bias in tree AGB estimations was considerably reduced when using VWWD predicted from m_1_ and m_4_ to convert tree volume to mass (B = 0.86% and 1.41% for m_1_ and m_4_, respectively). Including tree *DBH* in VWWD models further increased the model fit (∆AIC = −103.2 and −120.3 for m_2_ and m_5_ in comparison to m_1_ and m_4_, respectively) and reduced the bias in tree AGB estimates (B = 0.75% and 1.19% for m_2_ and m_5_, respectively; Figs. S3C,D and 2C,D). Although several other tree structure predictors were significant (e.g., stem morphology index *Sm* in m_3_ and m_6_), they often led to modest improvements of model fit (for instance, ∆AIC = −26.7 and −18 for m_3_ and m_6_ with respect to m_2_ and m_5_, respectively). We therefore favor using models m_2_ or m_5_, depending on the availability of local WD data.

We evaluated the robustness of our models using a leave-one-out cross-validation procedure, where the models were calibrated on all sites except a focal site (corresponding to a country in this case) and were used to predict tree VWWD for that site. Overall, the cross-validation models proved robust with B < 5% and CVs only slightly higher than when focal sites were used in model calibration (Fig. S4). This suggests that WD correction models should be transposable to other sites.

The role of intraindividual WD gradients had not previously been evaluated when applying terrestrial LiDAR scanning to estimate AGB. The existing studies either exhibited unexplained systematic bias^10,52^ or neglected the influence of vertical WD variations by using WD obtained from the Global Wood Density database in both predicted and reference AGB data^52^. In this study, we showed that WD gradients were a large source of uncertainty in tree-level AGB estimations and that they propagated systematic errors (bias) across our datasets that were far from being negligible (c. 10%). As TLS technology will likely play an increasing role in the development of nondestructive AGB allometries^10,11,52^ and the direct assessment of AGB at the stand level (notably for the calibration of upcoming satellite sensors^53^), care should be taken not to let the gain in the precision of wood volume be offset by the use of biased WD estimates. Here, we followed and generalized a seminal study^39,41^ in which the approach differed significantly from previous approaches^45,46^ in proposing to collect WD samples at specific locations in trees that would presumably be representative of the mean tree WD. Instead, we leveraged the relationships between WD estimates that are currently available (e.g., from the Global Wood Density database), WD vertical variation patterns and data that are routinely collected during field inventories to develop correction models that can easily be implemented for standard scientific inventory data. These models substantially reduced the error of tree-level AGB estimations and virtually removed the bias across our datasets, and they therefore constitute an efficient solution to the challenges of estimating tree biomass from volumetric data.

The results presented here provide a unique perspective on the homology between radial and vertical WD gradients, on the spectrum of regeneration and survival strategies in young stages, and on the converging biomechanical constraints in the canopy of reiterated trees. These findings should guide the collection of further biomechanical^50^ and physiological information required to improve our functional understanding of dense tropical forests. Our findings will also contribute to the development of nondestructive, precise and accurate methodologies for biomass assessment in tropical forests, with concrete implications for national greenhouse gas accounting, carbon trading schemes and REDD + frameworks in tropical countries.

## Materials and Methods

### Study area

The study was carried out in central Africa within the second largest continuous area of tropical forest after Amazonia^54^. Field work took place between July 2015 and February 2017, and the investigations were based on a standard destructive sampling protocol applied in six countries (Cameroon: Cam, Central African Republic: RCA, Democratic Republic of Congo: DRC, Republic of Congo: RC, Republic of Equatorial Guinea: REG, Gabon: Gab and Congo: Con), with one sampling site per country^5^. Only one forest type was sampled per country except in the RC, where both monodominant stands of *Gilbertiodendron dewevrei* and so-called “transition forests”^55^ from evergreen to semideciduous types were sampled.

The study area encompassed broad environmental gradients. The altitude across sampling sites ranged from c. 50 m a.s.l. in REG to more than 650 a.s.l. in Cam. The mean annual temperature (M.A.T.) was lowest in Cameroon (23.6 °C) and highest in the DRC and REG (25.2 °C). Mean annual rainfall (M.A.R.) ranged from a low of 1 396 mm in Cam to a high of 2 699 mm in REG. Details of the geographical coordinates and environmental conditions of the sampling sites are provided in Fayolle *et al*.^5^.

### Data collection

#### Destructive data

Sampling scheme. A total of 822 individuals belonging to 51 species and 16 families were destructively sampled across the Congo basin (Table S3). *Pterocarpus soyauxii* with 51 individuals was the most sampled species, while *Julbernardia pellegriniana* was the least sampled with 3 individuals. Within each country/forest type, the most abundant species were determined based on forest inventory data (for forest management and exploitation planning) and field expertise^5^. From each set of abundant species, ~15 species were chosen for each site to cover the range of the species mean wood density found at those sites.

Field protocol. The protocol for destructive sampling has been detailed by Fayolle *et al*.^5^. Before tree felling, the diameter at breast height (*DBH*, in cm) was measured at a height of 130 cm (or 30 cm above any deformations) using a 5 m-diameter tape. Total tree height and trunk height (defined as the height up to the first major living branch) were measured using a hypsometer device (Haglöf Vertex IV). Tree crown radii were measured in each cardinal direction by combining a clinometer (to locate the vertical projection of a given crown extremity on the ground) and a tape (to measure the distance between the projected crown extremity and the trunk). The tree crown radius (*Cr*, in m) was defined as the average of the four radius measurements.

After felling, the trees were divided into six vertical compartments: (1) the stump (*Stu*), defined as the part of the tree below the felling point (c. 1 m); (2) the stem base (*Ste*_*b*_) if present, which corresponds to the lowest meters of the stem (above the stump) that may be affected by deformations (typically buttresses) and therefore separated from the commercial part of the stem by loggers; (3) the stem (*Ste*), corresponding to the well-conformed, commercial part of the tree trunk (up to the base of the first major living branch); and (4) large-, (5) medium- and (6) small-size branches (indicated as *LB*, *MB* and *SB*), corresponding to branches with a basal diameter greater than 20 cm, between 20 and 5 cm and less than 5 cm, respectively.

Apart from the tree stump, tree parts with basal diameters < 70 cm were directly weighted in the field. Larger tree parts were divided into 1–2 m-long consecutive logs, and their volumes were estimated by approximating logs’ geometry as truncated cones.

In each compartment, two opposite wedge-shaped wood samples (30 to 50 mm thick) spanning the pith to the bark were taken to determine wood specific gravity. To reduce water losses, wood samples were sealed in plastic bags after extraction in the field and brought to the laboratory.

Laboratory analyses. For each wood sample, *I*, of compartment *c*, the fresh mass (*m*_*fi*_, in g) was measured with an electronic scale with a capacity of 6.2 kg and precision of 0.01 g, and the fresh volume (*v*_*fi*_, in cm^3^) was obtained following the Archimedes principle. Samples were then oven dried at 105 °C for two to three days until a constant dry mass was obtained (*m*_*di*_, in g). From those measurements, the basic wood density ($W{D}_{i}={m}_{di}/{v}_{fi}$, in g.cm^−3^), moisture content $(M{C}_{i}=({m}_{di}-{m}_{fi})/{m}_{fi})$ and fresh biomass-to-volume ratio ($B{V}_{i}={v}_{fi}/{m}_{fi}$, in cm^3^.g^−1^) of the samples were computed and averaged by compartment to obtain *WD*_*c*_, *MC*_*c*_ and *BV*_*c*_.

Volume and biomass estimations. The gold-standard for estimating total tree volume is by water-displacement method (i.e. Archimedes principle) of the entire tree. Similarly, direct weighting of the entire tree would provide the most reliable estimation of total tree mass. In practice, however, a combination of direct and indirect estimation methods are used due to logistical constraints, entailing unquantified uncertainties on tree volume and mass estimations. Here, the volume of logs that could not be weighted in the field (i.e., with a diameter ≥ 70 cm) was estimated considering logs as truncated cones^56^. For stumps with irregular shapes, the volume was computed using height measured in the field and the cross-sectional area computed from georeferenced photographs (see Fayolle *et al*.^5^ for details). Fresh volume and fresh biomass estimates were converted to dry biomass using the appropriate compartment-level WD (i.e., *WD*_*c*_) and MC (i.e., *MC*_*c*_), respectively. For a given tree, summing all dry biomass estimates within a compartment provided the compartment’s “observed” biomass (*AGB*_*c*_), and summing all *AGB*_*c*_ values of the tree allowed the total tree “observed” AGB (*AGB*_*obs*_) to be computed. Field-measured fresh biomass was also converted to volume using the appropriate *BV*_*c*_, yielding compartment- and tree-level volumes (*V*_*c*_ and *V*_*obs*_, respectively). Converting *V*_*obs*_ to biomass using a single WD value such as the species average WD values found in the Global Wood Density database (hereafter, *WD*_*GWD*_^13,46^), produced an estimated dry biomass value (*AGB*_*est*_).

Volume-weighted wood density. Following Sagang *et al*.^41^, we computed the volume-weighted wood density (*VWWD*_*c*_, in g.cm^−3^) at the compartment level. For example,

1 $$VWW{D}_{c}=W{D}_{c}\times \frac{{V}_{c}}{{V}_{obs}}$$

At the individual tree level, an unbiased estimator of wood-specific gravity is the sum of *VWWD*_*c*_, which we hereafter refer to as *VWWD*. In practice, multiplying *V*_*obs*_ by *VWWD* yields the reference tree biomass, *AGB*_*obs*_.

#### Terrestrial LiDAR data

The terrestrial laser scanning (TLS) data used in this study derive from^10^ and come from 58 trees sampled at the Cameroon field site. The trees were scanned with a Leica C10 Scanstation prior to being destructively sampled within the framework of the PREREDD + project. A minimum of three scans were performed around each targeted tree to obtain a sufficient point cloud density for high-quality reconstruction of tree structure. Quantitative structure models (QSMs) were generated using the *SimpleTree* algorithm^57^, and the QSMs were manually refined using dedicated software (refer to Momo *et al*.^10^ for details).

#### Species functional traits

We used COFOR-traits, a database of central African species functional traits extracted from local floras, academic papers and unpublished theses^58–60^, to assign the species regeneration guild (pioneer, light demanding nonpioneer and shade tolerant) to each species (Table S1).

### Statistical analyses

#### Characterizing the vertical variation patterns of wood-specific gravity

We compiled compartment-level wood density measurements (*WD*_*c*_) for all 822 trees in a table with individual trees in rows and the six compartments (*Stu*, *Ste*_*b*_, *Ste*, *LB*, *MB*, *SB*) in columns. Each table line therefore represented a vertical variation profile of wood density from the tree stump to the finest branches for a given tree. To systematically compare these profiles across trees, the *WD*_*c*_ table was subjected to principal component analysis (PCA). We centered the *WD*_*c*_ table by lines prior to re-performing the PCA to discard intertree variation in overall WD while focusing on the sole vertical gradient.

#### Taxonomic analysis

We recovered a phylogenetic tree for 49 of our 51 species using the PHYLOMATIC v.3 utility^61^ based on the Davies *et al*.^62^ phylogenetic hypothesis for relationships among angiosperm families, with polytomies applied within most families and genera. To evaluate whether the species vertical profile in WD was evolutionarily conserved, we determined the observed phylogenetic signal using Blomberg’s K on the basis of the species mean PCA1 score. Blomberg’s K values vary between zero and > 1, with values close to zero indicating no phylogenetic signal and values close to 1 indicating trait evolution according to Brownian motion (i.e., random walk divergence in species similarity). To evaluate significance, we used a rank-based P-value with Blomberg’s K for 999 randomizations of phylogeny tips.

#### Accounting for the vertical variation of wood density when converting tree volume to biomass

Converting tree volume (derived from either destructive data or QSMs) to biomass requires the use of a WD value that may not be representative of the whole-tree WD, leading to biased tree AGB estimation. We computed *AGB*_*est*_ using tree-level WD recorded approximately at breast height (*WD*_*Stu*_) and species mean WD from the literature (i.e., *WD*_*GWD*_), both of which are of potential interest for the routine conversion of TLS-derived tree volume to biomass. While both *WD*_*Stu*_ and *WD*_*GWD*_ neglect WD vertical variation within trees, *WD*_*Stu*_ accounts for site-level and individual tree-level variations and is therefore expected to provide more accurate *AGB*_*est*_ values. We characterized the deviations of *AGB*_*est*_ from *AGB*_*obs*_ across trees using a measure of bias (B) and a measure of total error (CV). B was calculated as the average of tree-level relative errors, *b*_*i*_:

$${b}_{i}=\frac{AG{B}_{est.i}-AG{B}_{obs.i}}{AG{B}_{obs.i}}\ast 100$$

where *AGB*_*est.i*_ and *AGB*_*obs.i*_ are the estimated and observed AGB of tree *i*.

The coefficient of variation (CV) was defined as follows:

$$RSE=\sqrt{\frac{1}{N}\ast \mathop{\sum }\limits_{i=1}^{N}{(AG{B}_{est.i}-AG{B}_{obs.i})}^{2}},$$

where *N* is the total number of trees,

$$MAG{B}_{obs}=\frac{1}{N}\ast \mathop{\sum }\limits_{i=1}^{N}AG{B}_{obs.i}$$

$$CV=\,\frac{RSE}{MAG{B}_{obs}}$$

We developed simple linear models to predict the unbiased estimator of whole-tree WD (i.e., VWWD) based on tree wood (*WD*_*Stu*_, *WD*_*GWD*_) and structure (*DBH*, stem morphology*)* parameters. The stem morphology, *Sm*, is defined as the ratio of trunk height to total tree height^41^. We evaluated and compared model performance using classical fit metrics (adjusted R², RSE, AIC) as well as B and CV for tree biomass, where *AGB*_*est*_ was computed using the models’ VWWD predictions. All analyses were performed with R statistical software 3.4.3 (65).

## Supplementary information

Supplementary figures and tables.

## Data Availability

The wood density data used in this study is available at 10.23708/8DUWUC.
