# Supplementary material for: Leveraging Signatures of Plant Functional Strategies in Wood Density Profiles of African Trees to Correct Mass Estimations From Terrestrial Laser Data
Source: Sci Rep. 2020 Feb 6;10:2001. doi: 10.1038/s41598-020-58733-w (PMC7005061; doi:10.1038/s41598-020-58733-w)
Supplement: Supplementary file 1 — Supplementary figures and tables. [file 41598_2020_58733_MOESM1_ESM.docx]

# Supplementary figures and tables

**Title.**

Leveraging signatures of plant functional strategies in wood density profiles of African trees to correct mass estimations from terrestrial laser data

**Authors.**

Stéphane Takoudjou Momo^1,2,*^ , Pierre Ploton^2,*^, Olivier Martin-Ducup ^2^, Romain Lehnebach^3^, Claire Fortunel^2^, Le Bienfaiteur Takougoum Sagang^1,2^, Faustin Boyemba^4^, Pierre Couteron^2^, Adeline Fayolle^5^, Moses Libalah^1^, Joel Loumeto^6^, Vincent Medjibe^7^, Alfred Ngomanda^8^, Diosdado Obiang^9^, Raphaël Pélissier^2^, Vivien Rossi^1,7,11^, Olga Yongo^10^, PREREDD collaborators^12^, Bonaventure Sonké^1^, Nicolas Barbier^2, †^

Figure S1. Relationship between tree WD measured at the stump level (panel A) or species average WD extracted from the Global Wood Density database (panel B) and vertical variation pattern of tree WD, defined as tree score on PCA axis 1. Illustrative species are highlighted following the color code of Figure 1 (i.e. *Pentaclethra macrophylla*, *Gilbertiodendron dewevrei* and *Poga oleosa* in dark red, light green and purple, respectively). The dotted black line represents the fit of a simple linear model.


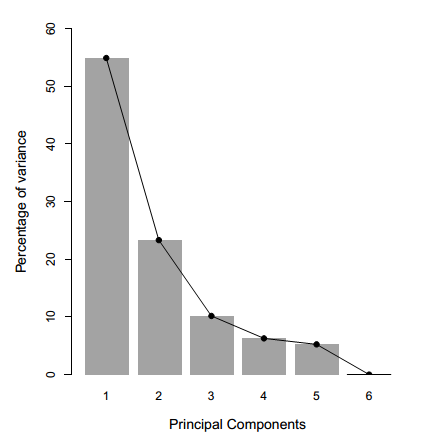


Figure S2. Histogram of eigenvalues associated to the Principal Component Analysis in Fig. 1A.


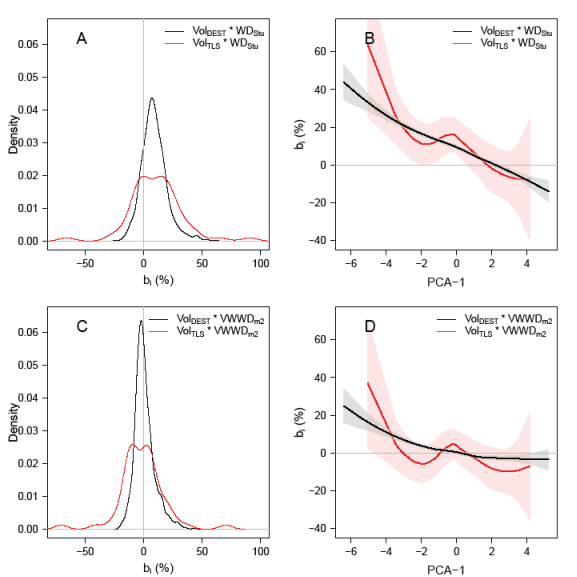


Figure S3. Bias in volume to mass conversion due to vertical WD gradients induced by the use of WD_Stu_. (A) Density plot of relative errors ($b_{i}$) on tree biomass estimation computed from combinations of tree volume (destructive data: black line, n = 822 ; LiDAR data: red line, n = 58) and tree wood density at the stump level (${WD}_{Stu}$). (B) Relationship between tree relative vertical WD profile (characterized by tree PCA1 score) and $b_{i}$ of panel A. Bias is reduced when using a tree-level estimate of WD, the volume-weighted wood density (${VWWD}_{m2}$): (C) Density plot of $b_{i}$ on tree biomass estimation computed from combinations of tree volume and volume-weighted wood density ($VWWD$) predicted from ${WD}_{Stu}$ and tree DBH (model 2, see Table 2 in the main text). (D) Relationship between tree relative vertical WD profile and $b_{i}$ of panel C.


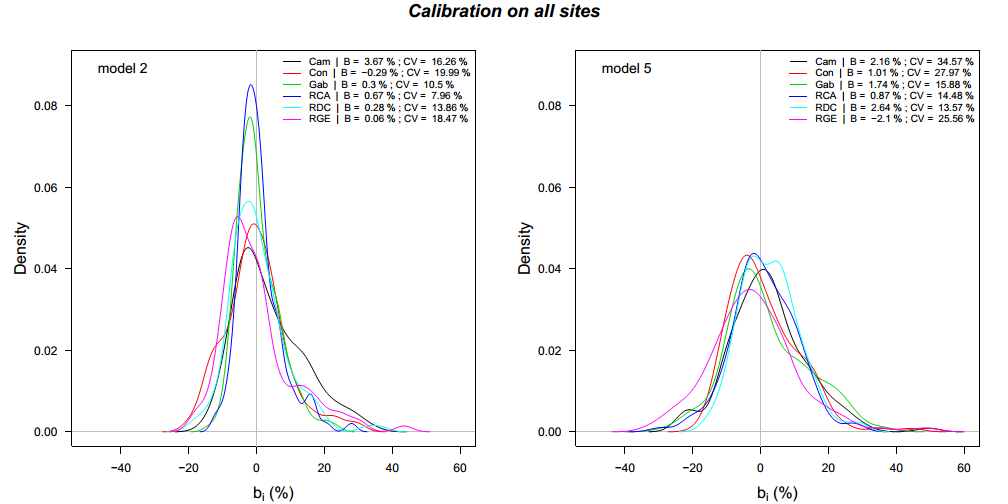


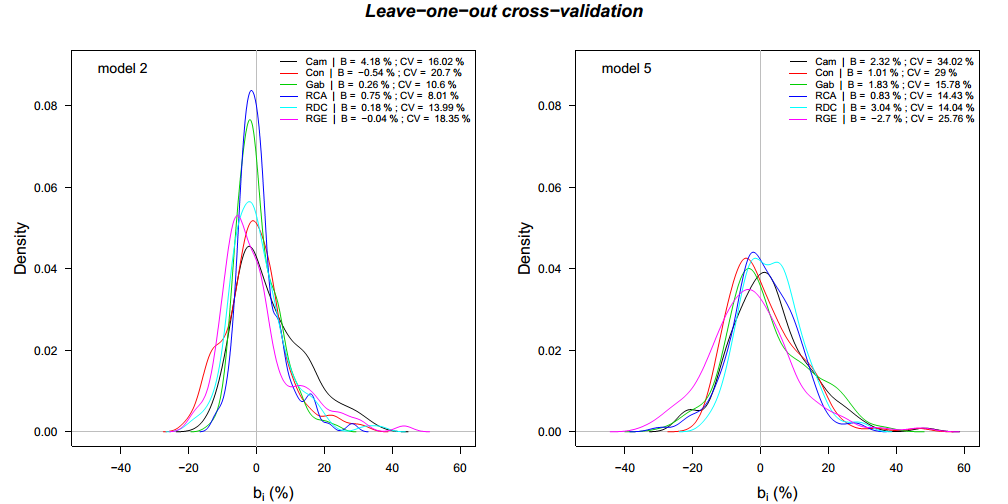


Figure S4: Site-level bias (B) and total error (CV) on AGB_est_ computed from tree volume and tree VWWD predicted from models 2 and 5. In top panels, models were calibrated on all sites. In bottom panels, models were calibrated following a leave-one-out cross-validation procedure, with B and CV computed when sites were not used in models’ calibration.

Figure S5: Bias in AGB estimations from allometric models due to vertical WD gradients. Tree AGB estimations were computed with the pantropical AGB model (Chave et al., 2014), using either tree WD taken at the stump-level (WD_Stu_) or species mean wood density extracted from the Global Wood Density database (WD_GWD_). Local regressions (loess function) are used to represent relationships between relative errors on tree AGB predictions (in %) and tree relative vertical WD profile (characterized by tree PCA1 score, panel A) or WD_GWD_ (panel B). AGB predictions from the allometric model (maroon and blue lines) and from a direct volume-to-AGB conversion (black line) show similar error patterns along PCA1. The PCA1 metric explained 8.4% of the allometric model’s prediction error.

Table S1 : Species characteristics: regeneration guild (Pioneer: P, non-pioneer light demanding: NPLD and shade tolerant: ST), phenology (deciduous: Dec and evergreen: Ever), dispersal syndrome (autochore: Auto, Anemochore: anemo and zoochore: Zoo), mean species score of PCA1 and 2 (with standard deviation between brackets), mean species WD per tree compartments with standard deviation between brackets.

| Species code | Guild | Pheno | Disper | PCA1 | PCA2 | WD_Stu_ | WD_Steb_ | WD_Ste_ | WD_SB_ | WD_MB_ | WD_LB_ | n |
| --- | --- | --- | --- | --- | --- | --- | --- | --- | --- | --- | --- | --- |
| Alsbo | P | Dec | Anemo | 1.28 (0.7) | -0.38 (0.47) | 0.392 (0.037) | 0.373 (0.053) | 0.375 (0.046) | 0.392 (0.047) | 0.382 (0.068) | 0.373 (0.064) | 9 |
| Annch | ST | Ever | Zoo | 0.46 (0.85) | -0.05 (0.59) | 0.507 (0.053) | 0.453 (0.042) | 0.446 (0.03) | 0.41 (0.063) | 0.45 (0.024) | 0.451 (0.027) | 5 |
| Anoma | ST | Ever | Zoo | 1.39 (0.5) | -0.02 (0.6) | 0.327 (0.02) | 0.331 (0.017) | 0.328 (0.022) | 0.328 (0.064) | 0.339 (0.019) | 0.331 (0.015) | 13 |
| Auckl | P | Ever | Anemo | 1.71 (1.08) | -0.61 (0.73) | 0.429 (0.062) | 0.395 (0.067) | 0.385 (0.065) | 0.423 (0.074) | 0.425 (0.076) | 0.422 (0.073) | 35 |
| Autco | NPLD | Dec | Zoo | -1.37 (0.78) | 0.08 (0.85) | 0.785 (0.038) | 0.779 (0.04) | 0.698 (0.039) | 0.613 (0.038) | 0.656 (0.049) | 0.7 (0.059) | 12 |
| Baple | ST | Ever | Auto | -0.74 (0.77) | 1.82 (1.47) | 0.808 (0.055) | 0.809 (0.05) | 0.797 (0.058) | 0.643 (0.064) | 0.729 (0.034) | 0.788 (0.066) | 6 |
| Brala | NPLD | Dec |  | 2.01 (0.56) | -1.17 (0.92) | 0.479 (0.031) | 0.454 (0.041) | 0.43 (0.045) | 0.501 (0.024) | 0.494 (0.027) | 0.47 (0.037) | 9 |
| Calhe | NPLD |  | Auto | -1.73 (1.37) | -1.37 (2.71) | 0.82 (0.114) | 0.722 (0.123) | 0.647 (0.096) | 0.617 (0.149) | 0.622 (0.091) | 0.639 (0.089) | 7 |
| Cansc | NPLD | Dec | Zoo | 2.4 (1.04) | -0.49 (0.77) | 0.486 (0.066) | 0.405 (0.059) | 0.411 (0.037) | 0.441 (0.06) | 0.503 (0.057) | 0.487 (0.045) | 8 |
| Celad | P | Dec | Zoo | -0.73 (0.79) | -1.1 (0.75) | 0.667 (0.053) | 0.635 (0.042) | 0.561 (0.057) | 0.542 (0.052) | 0.559 (0.061) | 0.551 (0.065) | 9 |
| Cylga | ST | Dec | Anemo | -2.6 (1.05) | -0.16 (1.99) | 0.836 (0.091) | 0.808 (0.093) | 0.708 (0.12) | 0.604 (0.052) | 0.631 (0.058) | 0.693 (0.095) | 19 |
| Cynha |  | Ever | Auto | 0.16 (0.73) | 0.28 (0.77) | 0.75 (0.042) | 0.696 (0.043) | 0.69 (0.04) | 0.632 (0.038) | 0.678 (0.03) | 0.695 (0.038) | 12 |
| Desgl |  | Ever | Anemo | -1.55 (1.87) | -0.48 (1.26) | 0.86 (0.032) | 0.852 (0.045) | 0.771 (0.064) | 0.695 (0.076) | 0.736 (0.082) | 0.736 (0.089) | 12 |
| Diapa | ST | Ever | Zoo | -1.79 (1.43) | 0.22 (0.87) | 0.808 (0.059) | 0.782 (0.038) | 0.717 (0.046) | 0.609 (0.083) | 0.657 (0.047) | 0.696 (0.057) | 13 |
| Dubma | NPLD | Ever | Zoo | -0.06 (1.1) | 1.28 (1.15) | 0.545 (0.065) | 0.561 (0.07) | 0.542 (0.048) | 0.429 (0.119) | 0.507 (0.073) | 0.539 (0.049) | 7 |
| Entca | NPLD | Dec | Anemo | 1.06 (1.72) | -0.56 (1.15) | 0.573 (0.036) | 0.576 (0.039) | 0.531 (0.053) | 0.548 (0.064) | 0.559 (0.066) | 0.56 (0.071) | 9 |
| Entcy | NPLD | Dec | Anemo | 0.45 (1.23) | 0.07 (0.8) | 0.569 (0.044) | 0.563 (0.045) | 0.544 (0.049) | 0.513 (0.05) | 0.53 (0.051) | 0.541 (0.056) | 40 |
| Eriob | ST | Dec | Zoo | -0.98 (1.62) | -0.85 (1.56) | 0.616 (0.052) | 0.611 (0.031) | 0.513 (0.074) | 0.482 (0.057) | 0.503 (0.074) | 0.519 (0.088) | 9 |
| Eryiv | NPLD | Ever | Auto | -0.93 (1.35) | 0.56 (0.88) | 0.774 (0.059) | 0.72 (0.098) | 0.691 (0.073) | 0.592 (0.058) | 0.646 (0.064) | 0.693 (0.084) | 10 |
| Erysu | NPLD | Dec | Auto | -1.06 (1.03) | 0.22 (1.52) | 0.811 (0.058) | 0.755 (0.055) | 0.73 (0.061) | 0.647 (0.103) | 0.675 (0.05) | 0.711 (0.065) | 22 |
| Gilde | ST | Ever | Auto | 0.93 (0.67) | 1.2 (0.55) | 0.645 (0.037) | 0.664 (0.032) | 0.67 (0.03) | 0.597 (0.029) | 0.649 (0.029) | 0.675 (0.04) | 39 |
| Guace | ST | Ever | Zoo | -0.41 (0.48) | -0.15 (0.52) | 0.605 (0.056) | 0.59 (0.057) | 0.547 (0.062) | 0.497 (0.038) | 0.523 (0.048) | 0.534 (0.052) | 6 |
| Irvgr | NPLD | Dec | Zoo | -1.43 (0.6) | -0.06 (0.73) | 0.813 (0.039) | 0.801 (0.034) | 0.73 (0.045) | 0.646 (0.03) | 0.684 (0.041) | 0.711 (0.058) | 8 |
| Julpe | ST | Ever | Auto | -0.13 (2.43) | 0.19 (1.54) | 0.656 (0.037) | 0.751 (0.236) | 0.652 (0.018) | 0.598 (0.017) | 0.641 (0.02) | 0.661 (0.012) | 3 |
| Khaan | NPLD | Dec | Anemo | 1.02 (0.96) | 0.29 (0.36) | 0.552 (0.039) | 0.519 (0.035) | 0.521 (0.034) | 0.488 (0.039) | 0.53 (0.044) | 0.538 (0.051) | 8 |
| Klaga | NPLD | Dec | Zoo | 0.07 (1.68) | 1.14 (0.7) | 0.782 (0.172) | 0.789 (0.166) | 0.794 (0.137) | 0.7 (0.103) | 0.742 (0.098) | 0.769 (0.105) | 5 |
| Lopal | P | Dec | Anemo | -1.8 (1.34) | 0.87 (1.19) | 0.875 (0.048) | 0.867 (0.037) | 0.808 (0.053) | 0.662 (0.088) | 0.743 (0.076) | 0.784 (0.071) | 34 |
| Lovtr | NPLD | Dec | Anemo | 0.17 (1.27) | 0.48 (0.73) | 0.633 (0.064) | 0.609 (0.067) | 0.594 (0.051) | 0.537 (0.013) | 0.569 (0.023) | 0.603 (0.038) | 6 |
| Macba | P | Ever | Auto | 2.02 (0.63) | -0.87 (0.48) | 0.381 (0.05) | 0.349 (0.036) | 0.345 (0.033) | 0.41 (0.047) | 0.388 (0.053) | 0.379 (0.041) | 12 |
| Manal | NPLD | Dec | Anemo | 0.15 (0.69) | 0.85 (0.7) | 0.534 (0.026) | 0.526 (0.022) | 0.515 (0.024) | 0.43 (0.029) | 0.489 (0.015) | 0.513 (0.035) | 7 |
| Manma | SB | Ever | Zoo | -1.35 (0.52) | -0.25 (0.44) | 0.797 (0.023) | 0.784 (0.033) | 0.715 (0.027) | 0.644 (0.031) | 0.67 (0.024) | 0.692 (0.029) | 11 |
| Milex | P | Dec | Zoo | 0.14 (0.75) | -0.4 (0.93) | 0.593 (0.056) | 0.532 (0.05) | 0.506 (0.058) | 0.48 (0.045) | 0.51 (0.044) | 0.518 (0.06) | 21 |
| Onggo | NPLD | Ever | Zoo | -0.11 (0.52) | 0.88 (0.47) | 0.713 (0.02) | 0.702 (0.02) | 0.686 (0.027) | 0.596 (0.022) | 0.651 (0.026) | 0.685 (0.024) | 7 |
| Penma | NPLD | Ever | Auto | -2.91 (1.05) | 0.66 (1.2) | 0.848 (0.06) | 0.818 (0.066) | 0.75 (0.073) | 0.586 (0.049) | 0.65 (0.044) | 0.7 (0.059) | 12 |
| Perel | NPLD | Dec | Anemo | -0.86 (1.07) | 0.05 (0.87) | 0.727 (0.043) | 0.664 (0.062) | 0.635 (0.058) | 0.559 (0.018) | 0.602 (0.023) | 0.626 (0.032) | 9 |
| Petma | NPLD | Dec | Anemo | -0.76 (1.23) | 0.37 (1.07) | 0.621 (0.042) | 0.622 (0.062) | 0.573 (0.049) | 0.487 (0.072) | 0.535 (0.056) | 0.559 (0.059) | 50 |
| Plaaf |  | Ever | Zoo | 0.39 (0.55) | 0.28 (0.47) | 0.593 (0.058) | 0.588 (0.06) | 0.569 (0.058) | 0.512 (0.052) | 0.566 (0.039) | 0.561 (0.054) | 12 |
| Pogol | ST |  | Zoo | 3.32 (0.95) | -0.46 (0.36) | 0.413 (0.024) | 0.404 (0.023) | 0.432 (0.03) | 0.522 (0.03) | 0.489 (0.041) | 0.481 (0.045) | 7 |
| Polsu | ST | Ever | Zoo | -0.96 (0.76) | -0.55 (0.79) | 0.654 (0.048) | 0.673 (0.038) | 0.603 (0.04) | 0.549 (0.027) | 0.575 (0.022) | 0.556 (0.047) | 12 |
| Priox | NPLD | Dec | Anemo | 1.95 (0.54) | -0.48 (0.57) | 0.534 (0.047) | 0.522 (0.045) | 0.529 (0.042) | 0.573 (0.045) | 0.555 (0.047) | 0.543 (0.043) | 10 |
| Pteso | NPLD | Dec | Anemo | -0.88 (1.83) | -0.68 (1.57) | 0.664 (0.053) | 0.648 (0.074) | 0.564 (0.083) | 0.524 (0.063) | 0.553 (0.079) | 0.568 (0.089) | 51 |
| Pycan | NPLD | Ever | Zoo | 0.66 (1.52) | -0.08 (1.1) | 0.439 (0.038) | 0.427 (0.041) | 0.407 (0.052) | 0.379 (0.046) | 0.413 (0.078) | 0.407 (0.066) | 54 |
| Riche | P | Dec | Zoo | 3.23 (1.12) | -0.4 (0.56) | 0.276 (0.015) | 0.25 (0.019) | 0.283 (0.032) | 0.352 (0.04) | 0.354 (0.065) | 0.328 (0.051) | 20 |
| Santr | ST | Ever | Zoo | 0.11 (0.89) | 0.08 (0.91) | 0.591 (0.035) | 0.57 (0.039) | 0.537 (0.022) | 0.49 (0.054) | 0.53 (0.029) | 0.546 (0.019) | 9 |
| Scoze | ST | Ever | Auto | -0.17 (0.32) | 1.12 (0.7) | 0.68 (0.02) | 0.669 (0.021) | 0.655 (0.023) | 0.543 (0.036) | 0.623 (0.023) | 0.653 (0.025) | 7 |
| Scyma | NPLD | Ever | Zoo | -0.21 (0.81) | 0.12 (0.93) | 0.478 (0.033) | 0.466 (0.041) | 0.433 (0.048) | 0.377 (0.038) | 0.408 (0.038) | 0.424 (0.034) | 12 |
| Staka | ST | Ever | Zoo | -2.02 (0.82) | 0.13 (1.13) | 0.717 (0.063) | 0.721 (0.079) | 0.648 (0.067) | 0.539 (0.055) | 0.574 (0.055) | 0.601 (0.074) | 42 |
| Strgr | ST | Ever | Zoo | 0.01 (0.29) | 0.55 (0.63) | 0.653 (0.026) | 0.654 (0.025) | 0.642 (0.031) | 0.568 (0.035) | 0.61 (0.031) | 0.615 (0.029) | 8 |
| Tersu | P | Dec | Anemo | 1.55 (1.41) | -0.36 (1.11) | 0.511 (0.044) | 0.471 (0.043) | 0.477 (0.052) | 0.495 (0.073) | 0.504 (0.078) | 0.495 (0.08) | 28 |
| Trisc | P | Dec | Anemo | 1.46 (1.54) | -0.37 (1.14) | 0.425 (0.048) | 0.391 (0.048) | 0.369 (0.047) | 0.377 (0.05) | 0.423 (0.066) | 0.429 (0.086) | 20 |
| Xylae | P | Ever | Zoo | 1.39 (0.76) | -0.3 (0.95) | 0.571 (0.053) | 0.53 (0.065) | 0.53 (0.068) | 0.541 (0.047) | 0.555 (0.062) | 0.553 (0.065) | 26 |

Table S2 : Results of Tukey’s HSD post-hoc test on tree PCA1 score by life strategy guild (pioneer: P, non-pioneer light-demanding: NPLD and shade tolerant: ST).

| Linear Hypotheses | Estimate | Std Error | t value | P |
| --- | --- | --- | --- | --- |
| NPLD - P == 0 | -1,195 | 0,146 | -8,160 | < 0,0001 |
| ST - P == 0 | -1,680 | 0,165 | -10,167 | < 0,0001 |
| ST - NPLD == 0 | -0,485 | 0,149 | -3,256 | 0,0034 |

Table S3: Description of sampled species: number of trees per species (n_tree_), range of the tree diameter at breast height (DBH, in cm), mean and standard deviation of i) wood density derived from the Global Wood Density database (WD_GWD_ in g.cm^-3^), ii) wood specific gravity determined in the stump compartment (WD_Stu_ in g.cm^-3^) and iii) volume-weighted wood density (VWWD in g.cm^-3^).

| Family | Species | Code | n_tree_ | DBH | WD_GWD_ | WD_Stu_ | VWWD |
| --- | --- | --- | --- | --- | --- | --- | --- |
|  |  |  |  | (min – max) |  |  |  |
| Anisophylleaceae | *Poga oleosa* | Pogol | 7 | 24 - 130 | 0.393 (0.054) | 0.413 (0.024) | 0.444 (0.029) |
| Annonaceae | *Annickia chlorantha* | Annch | 5 | 11.3 - 51 | 0.437 (0.048) | 0.507 (0.053) | 0.445 (0.033) |
|  | *Anonidium mannii* | Anoma | 13 | 18.7 - 71.8 | 0.291 (0.009) | 0.327 (0.02) | 0.329 (0.019) |
|  | *Polyalthia suaveolens* | Polsu | 12 | 14.5 - 51 | 0.695 (NA) | 0.654 (0.048) | 0.593 (0.028) |
|  | *Xylopia aethiopica* | Xylae | 26 | 15 - 76.7 | 0.442 (0.115) | 0.571 (0.053) | 0.539 (0.061) |
| Apocynaceae | *Alstonia boonei* | Alsbo | 9 | 16 - 128.75 | 0.321 (0.064) | 0.392 (0.037) | 0.379 (0.046) |
| Burseraceae | *Aucoumea klaineana* | Auckl | 35 | 14 - 169.3 | 0.378 (0.054) | 0.429 (0.062) | 0.405 (0.066) |
|  | *Canarium schweinfurthii* | Cansc | 8 | 20.8 - 160.5 | 0.409 (0.062) | 0.486 (0.066) | 0.445 (0.044) |
|  | *Santiria trimera* | Santr | 9 | 12.3 - 52.5 | 0.546 (0.041) | 0.591 (0.035) | 0.543 (0.018) |
| Cannabaceae | *Celtis adolfi-friderici* | Celad | 9 | 17.7 - 70.5 | 0.581 (0.064) | 0.667 (0.053) | 0.568 (0.051) |
| Combretaceae | *Terminalia superba* | Tersu | 28 | 13 - 113.5 | 0.459 (0.064) | 0.511 (0.044) | 0.484 (0.051) |
| Euphorbiaceae | *Macaranga barteri* | Macba | 12 | 14.8 - 53.5 | 0.389 (0.053) | 0.381 (0.05) | 0.366 (0.036) |
|  | *Plagiostyles africana* | Plaaf | 12 | 17.1 - 51.5 | 0.741 (0.011) | 0.593 (0.058) | 0.565 (0.053) |
|  | *Ricinodendron heudelotii* | Riche | 20 | 14.5 - 126 | 0.211 (0.018) | 0.276 (0.015) | 0.297 (0.034) |
| Fabaceae | *Baphia leptobotrys* | Baple | 6 | 14.5 - 67 | 0.701 (0.246) | 0.808 (0.055) | 0.759 (0.041) |
|  | *Brachystegia laurentii* | Brala | 9 | 11.7 - 146.5 | 0.5 (0.039) | 0.479 (0.031) | 0.451 (0.037) |
|  | *Calpocalyx heitzii* | Calhe | 7 | 18 - 88 | 0.727 (0.054) | 0.82 (0.114) | 0.649 (0.097) |
|  | *Cylicodiscus gabunensis* | Cylga | 19 | 13.5 - 159.5 | 0.79 (0.079) | 0.836 (0.091) | 0.7 (0.1) |
|  | *Cynometra hankei* | Cynha | 12 | 17.8 - 112 | 0.841 (0.032) | 0.75 (0.042) | 0.687 (0.032) |
|  | *Dialium pachyphyllum* | Diapa | 13 | 16 - 129 | 0.923 (0.039) | 0.808 (0.059) | 0.705 (0.041) |
|  | *Erythrophleum ivorense* | Eryiv | 10 | 17.5 - 172 | 0.774 (0.06) | 0.774 (0.059) | 0.687 (0.065) |
|  | *Erythrophleum suaveolens* | Erysu | 22 | 16.6 - 120.5 | 0.873 (0.092) | 0.811 (0.058) | 0.72 (0.054) |
|  | *Gilbertiodendron dewevrei* | Gilde | 39 | 11.7 - 158 | 0.707 (0.023) | 0.645 (0.037) | 0.663 (0.027) |
|  | *Julbernardia pellegriniana* | Julpe | 3 | 80 - 108.6 | 0.675 (0.072) | 0.656 (0.037) | 0.652 (0.019) |
|  | *Pentaclethra macrophylla* | Penma | 12 | 11.5 - 112 | 0.841 (0.068) | 0.848 (0.06) | 0.717 (0.06) |
|  | *Pericopsis elata* | Perel | 9 | 14.8 - 158.5 | 0.639 (0.041) | 0.727 (0.043) | 0.629 (0.038) |
|  | *Prioria oxyphylla* | Priox | 10 | 14.8 - 133.3 | 0.57 (0.052) | 0.534 (0.047) | 0.537 (0.037) |
|  | *Pterocarpus soyauxii* | Pteso | 51 | 11.6 - 144 | 0.658 (0.072) | 0.664 (0.053) | 0.571 (0.074) |
|  | *Scorodophloeus zenkeri* | Scoze | 7 | 15.8 - 97.5 | 0.724 (0.088) | 0.68 (0.02) | 0.642 (0.014) |
| Irvingiaceae | *Desbordesia glaucescens* | Desgl | 12 | 14.5 - 170 | 0.915 (0.066) | 0.86 (0.032) | 0.768 (0.061) |
|  | *Irvingia grandifolia* | Irvgr | 8 | 10.3 - 134 | 0.801 (0.02) | 0.813 (0.039) | 0.726 (0.034) |
|  | *Klainedoxa gabonensis* | Klaga | 5 | 52 - 102 | 0.926 (0.103) | 0.782 (0.172) | 0.77 (0.123) |
| Lecythidaceae | *Petersianthus macrocarpus* | Petma | 50 | 12.5 - 115 | 0.677 (0.057) | 0.621 (0.042) | 0.57 (0.046) |
| Malvaceae | *Duboscia macrocarpa* | Dubma | 7 | 26.5 - 120.3 | 0.508 (0.135) | 0.545 (0.065) | 0.522 (0.053) |
|  | *Eribroma oblongum* | Eriob | 9 | 17.8 - 100.8 | 0.638 (0.034) | 0.616 (0.052) | 0.522 (0.065) |
|  | *Mansonia altissima* | Manal | 7 | 19 - 74.56 | 0.564 (0.035) | 0.534 (0.026) | 0.51 (0.021) |
|  | *Triplochiton scleroxylon* | Trisc | 20 | 13.5 - 208 | 0.335 (0.037) | 0.425 (0.048) | 0.39 (0.047) |
| Meliaceae | *Entandrophragma candollei* | Entca | 9 | 15 - 173 | 0.574 (0.071) | 0.573 (0.036) | 0.542 (0.051) |
|  | *Entandrophragma cylindricum* | Entcy | 40 | 15.2 - 169.42 | 0.572 (0.037) | 0.569 (0.044) | 0.544 (0.043) |
|  | *Guarea cedrata* | Guace | 6 | 16.5 - 70.5 | 0.527 (0.017) | 0.605 (0.056) | 0.542 (0.056) |
|  | *Khaya anthotheca* | Khaan | 8 | 12.1 - 113 | 0.491 (0.043) | 0.552 (0.039) | 0.526 (0.035) |
|  | *Lovoa trichilioides* | Lovtr | 6 | 45.5 - 107.6 | 0.455 (0.04) | 0.633 (0.064) | 0.591 (0.044) |
| Moraceae | *Milicia excelsa* | Milex | 21 | 18.5 - 116 | 0.575 (0.061) | 0.593 (0.056) | 0.51 (0.052) |
| Myristicaceae | *Pycnanthus angolensis* | Pycan | 54 | 13 - 130 | 0.409 (0.065) | 0.439 (0.038) | 0.412 (0.045) |
|  | *Scyphocephalium mannii* | Scyma | 12 | 36.8 - 109.8 | 0.507 (0.088) | 0.478 (0.033) | 0.429 (0.032) |
|  | *Staudtia kamerunensis* | Staka | 42 | 12.6 - 119.3 | 0.797 (0.073) | 0.717 (0.063) | 0.639 (0.059) |
| Ochnaceae | *Lophira alata* | Lopal | 34 | 11 - 152 | 0.897 (0.056) | 0.875 (0.048) | 0.792 (0.054) |
| Olacaceae | *Ongokea gore* | Onggo | 7 | 36.1 - 72.5 | 0.749 (0.042) | 0.713 (0.02) | 0.679 (0.023) |
|  | *Strombosia grandifolia* | Strgr | 8 | 14.5 - 54.7 | 0.825 (0.065) | 0.653 (0.026) | 0.623 (0.029) |
| Sapotaceae | *Autranella congolensis* | Autco | 12 | 10.5 - 200 | 0.777 (0.087) | 0.785 (0.038) | 0.698 (0.034) |
|  | *Manilkara mabokeënsis* | Manma | 11 | 14.5 - 106 | 0.867 (0.041) | 0.797 (0.023) | 0.708 (0.023) |
